# Choosing between dual and three-drug antiretroviral therapy in aging and comorbid people with HIV: a narrative review

**DOI:** 10.3389/frph.2026.1805648

**Published:** 2026-06-22

**Authors:** Maria Vittoria Cossu, Maddalena Matone, Valentina Iannone, Chiara Fusetti, Valeria Colombo, Andrea Gori, Anna Lisa Ridolfo, Cristina Gervasoni

**Affiliations:** 1 ASST Fatebenefratelli Sacco, Milan, Italy; 2 Department of Infectious Diseases, ASST Fatebenefratelli Sacco University Hospital, Milan, Italy; 3 Department of Biomedical and Clinical Sciences “L. Sacco”, University of Milan, Milan, Italy; 4 Centre for Multidisciplinary Research in Health Science (MACH), Milan, Italy

**Keywords:** aging, antiretroviral therapy, comorbidities, dual therapy, HIV, polypharmacy, randomized controlled trials, real-world evidence

## Abstract

The widespread use of combination antiretroviral therapy has transformed HIV infection into a chronic condition, with life expectancy of people with HIV (PWH) approaching that of the general population when treatment is initiated early and virological suppression is maintained. As survival has improved, the demographic profile of the HIV population has shifted substantially, with a growing proportion of PWH in high-income countries now aged 50 years and older. In this context, the goals of HIV care extend beyond durable virological suppression to include long-term tolerability, management of multimorbidity, reduction of polypharmacy-related risks, and preservation of functional status and quality of life. Three-drug antiretroviral regimens (3DR) have long represented the standard of care due to their robust efficacy and high genetic barrier to resistance. However, in aging and comorbid PWH, lifelong exposure to multiple antiretroviral agents may increase the risk of cumulative organ toxicity—including renal, bone, and cardiovascular adverse effects—as well as clinically relevant drug–drug interactions with medications used to manage concomitant conditions. These considerations have driven interest in treatment simplification strategies, with dual antiretroviral therapy (2DR) emerging as the most clinically investigated approach. Current international guidelines endorse specific 2DR strategies on the basis of randomized controlled trials demonstrating non-inferior virological efficacy compared with 3DR in carefully selected populations. This narrative review integrates evidence from randomized trials and real-world observational studies to critically appraise the role of guideline-endorsed 2DR vs. 3DR in aging and comorbid PWH. We highlight clinical scenarios in which simplification may be advantageous, identify factors associated with reduced durability, and propose a patient-centred framework to support individualized antiretroviral therapy selection, one that extends beyond virological endpoints to incorporate the geriatric dimensions of contemporary HIV care.

## 1 Introduction

The widespread implementation of combination antiretroviral therapy (ART) has profoundly transformed HIV infection into a chronic, manageable condition, with life expectancy for people with HIV (PWH) approaching that of the general population when treatment is initiated early and durable virological suppression is achieved and maintained (1). As survival has improved over the past three decades, the epidemiology of HIV has shifted substantially: in high-income countries, more than half of PWH are now aged 50 years or older, with a rapidly increasing proportion aged 65 years and above (2). In line with commonly used definitions in HIV research, “older PWH” refers to individuals aged ≥50 years, acknowledging that chronological age alone does not fully capture biological aging, frailty, or multimorbidity; those aged ≥65 years represent a growing and clinically distinct subgroup deserving particular attention (3, 4). This demographic transition has fundamentally reshaped the goals of HIV care: beyond achieving the virological suppression, clinicians are increasingly called to integrate HIV management within a broader framework of multimorbidity, functional decline, and healthy aging.

Aging with HIV is associated with a growing burden of non-AIDS comorbidities, including cardiovascular disease, chronic kidney disease, metabolic disorders, bone disease with increased risk of osteoporosis and fractures, neurocognitive impairment, and malignancies (3). Several of these conditions occur earlier and with greater prevalence than in HIV-negative populations, a phenomenon often referred to as “accentuated” or “accelerated” aging, although its definitions and underlying mechanisms remain debated (5). The biological basis of this excess risk is multifactorial, involving persistent immune activation and dysfunction, gut barrier impairment, and microbial translocation, all contributing to chronic inflammation and immunosenescence despite sustained virological suppression. ART-related metabolic effects and adverse social determinants, including marginalization, mental health disorders, and substance use, may further amplify clinical vulnerability (6, 7). Consequently, the management of older PWH increasingly aligns with principles of geriatric medicine, requiring treatment decisions that balance efficacy, toxicity, drug–drug interactions, pill burden, and patient preferences.

Polypharmacy has become a defining feature of care for aging PWH. Observational cohorts consistently report that many older individuals receive five or more non-ART medications, substantially increasing the risk of drug–drug interactions (DDI), adverse drug events, and prescribing cascades (8). Although contemporary ART regimens are generally well tolerated, concerns persist regarding cumulative long-term exposure to agents associated with renal, bone, metabolic, neuropsychiatric, or cardiovascular toxicity (9). In this setting, the number and pharmacological profile of antiretroviral agents are no longer neutral background characteristics but clinically relevant variables.

With the improved potency, tolerability, and durability of newer antiretroviral agents and their higher barrier to resistance, interest has emerged in two-drug regimens (2DR) as a clinically relevant alternative to the established three-drug regimen (3DR) paradigm. A key rationale shared across 2DR strategies is the elimination of NRTIs associated with cumulative organ toxicity—in particular tenofovir disoproxil fumarate, linked to renal and bone injury, and abacavir, associated with increased cardiovascular risk. The possibility that simpler regimens may better sustain adherence over decades of treatment further supports this approach.

Based on evidence from Randomized controlled trials (RCTs), current HIV treatment guidelines endorse selected 2DR strategies as switch options in virologically suppressed individuals, and—for specific oral regimens—also as initial therapy in appropriately selected patients.

Nevertheless, translating trial-based evidence into routine clinical practice remains challenging. RCTs provide high internal validity but typically enroll selected populations, often excluding individuals with multimorbidity, advanced frailty, complex treatment histories—precisely the patients most commonly encountered in aging HIV care. In addition, trial participants benefit from intensive follow-up, structured adherence support, and protocol-driven monitoring, conditions that may not reflect routine care. Real-world observational studies, while subject to confounding and variability in monitoring intensity, capture this heterogeneity and provide complementary insight into how 2DR strategies perform outside controlled environments. Integrating both sources of evidence is therefore essential to inform individualized treatment decisions in aging and comorbid PWH for whom optimal treatment may depend as much on long-term safety and quality of life as on virological efficacy.

This narrative review integrates evidence from RCTs and real-world cohorts to inform clinical decision of when and for whom guideline-endorsed 2DR strategies represent an appropriate option in aging and comorbid people with HIV.

## 2 Methods

A literature search was performed in PubMed/MEDLINE and Embase for studies published up to December 2025, using combinations of the following terms: “HIV”, “dual therapy”, “two-drug regimen”, “dolutegravir/lamivudine”, “dolutegravir/rilpivirine”, “cabotegravir/rilpivirine”, “darunavir/lamivudine”, “darunavir/dolutegravir”, and “long-acting injectable antiretroviral”. Studies were eligible if they evaluated one or more guideline-endorsed or clinically investigated dual antiretroviral strategies—specifically dolutegravir/lamivudine, dolutegravir/rilpivirine, cabotegravir/rilpivirine, darunavir/ritonavir plus lamivudine, or darunavir/ritonavir plus dolutegravir—in virologically suppressed or treatment-naive adults with HIV. Priority was given to randomized controlled trials and large real-world cohort studies reporting outcomes of virological efficacy, safety, tolerability, or durability, with additional attention to data specifically addressing older or comorbid populations. Studies reporting exclusively on younger, treatment-naive cohorts without age- or comorbidity-stratified analyses were considered of lower priority. Additional references were identified through manual screening of bibliography lists and consultation of current international guidelines, including the European AIDS Clinical Society (EACS) and the US Department of Health and Human Services (DHHS) recommendations. The search and study selection were conducted between December 2025 and January 2026.

## 3 Guideline-endorsed 2DR oral therapy: evidence from RCTs and real-world studies

Current international guidelines endorse two integrase-based dual antiretroviral strategies: dolutegravir/lamivudine (DTG/3TC) and dolutegravir/rilpivirine (DTG/RPV). Both regimens share dolutegravir—a high-barrier integrase strand transfer inhibitor—as the anchor agent, while differing in eligibility criteria, resistance requirements, and pharmacological interactions. Additional dual strategies based on boosted protease inhibitors—specifically darunavir plus lamivudine (DRV/r or DRV/c + 3TC) and darunavir plus dolutegravir (DRV/r + DTG)—have also been investigated, primarily as nucleoside-sparing switch options. The evidence base for each strategy is reviewed below, integrating data from randomized controlled trials and real-world observational studies, with specific attention to findings relevant to older and comorbid PWH.

### 3.1 Evidence in controlled clinical trials

#### 3.1.1 Dolutegravir/lamivudine (DTG/3TC)

DTG/3TC is the only dual regimen currently recommended by international guidelines both as initial therapy in treatment-naive patients and as a switch option in virologically suppressed individuals, reflecting the breadth and maturity of its evidence base.

In treatment-naive adults, the pivotal evidence derives from the GEMINI-1 and GEMINI-2 trials, two identical phase III randomized, double-blind studies comparing DTG/3TC with DTG plus tenofovir disoproxil fumarate/emtricitabine (TDF/FTC). At 48 weeks, both trials demonstrated non-inferiority of DTG/3TC, with virological suppression rates of approximately 91%–93% in both arms (1, 2). These results were sustained through 144 weeks of follow-up, with no emergent resistance to either dolutegravir or lamivudine in the DTG/3TC arm, underscoring the high genetic barrier of the combination (3).

Importantly, DTG/3TC was associated with significantly better renal and bone safety parameters compared with the TDF-containing regimen—a finding of particular relevance in older treatment-naive patients with pre-existing renal impairment or reduced bone mineral density. Of note, the GEMINI trials excluded individuals with pre-treatment HIV-RNA above 500,000 copies/mL, hepatitis B coinfection, and documented resistance mutations, eligibility criteria that remain integral to guideline recommendations for use in naive patients.

As a switch strategy in virologically suppressed adults, DTG/3TC was evaluated in the TANGO trial, which assessed switching from TAF-based three-drug regimens, and in the SALSA trial, which included a broader range of prior regimens. Both studies demonstrated non-inferiority at 48 and 96 weeks, with virological suppression rates exceeding 90% and no confirmed virological failures with emergent resistance in the DTG/3TC arm (4–6). Together, these trials established DTG/3TC as a robust switch option in adults with stable virological suppression and no prior virological failure on lamivudine or emtricitabine.

With respect to older and comorbid populations, pre-specified subgroup analyses from GEMINI, TANGO, and SALSA generally showed consistent virological outcomes in individuals aged ≥50 years (7). However, patients with significant comorbidities, estimated glomerular filtration rate below 50 mL/min, or active hepatitis B coinfection were excluded from all these trials, limiting direct generalizability to the most clinically complex older PWH.

### 3.2 Real-world evidence

#### 3.2.1 Dolutegravir/lamivudine: real-world cohorts

Real-world evidence on DTG/3TC has expanded substantially since its guideline endorsement, with multiple observational cohorts confirming high rates of virological suppression ranging from 88 to 96% at 48 weeks in populations more heterogeneous than those enrolled in pivotal trials (8–10). In older and comorbid PWH, cohort studies have demonstrated comparable outcomes to younger patients, with switches frequently motivated by tolerability concerns and the need to reduce drug–drug interactions with cardiometabolic or anticoagulant therapies (11). Predictors of virological failure include low nadir CD4 count, prior lamivudine exposure with suspected resistance, and suboptimal adherence (12).

#### 3.2.2 Dolutegravir/rilpivirine: real-world cohorts

Real-world cohorts broadly confirm the virological efficacy of DTG/RPV demonstrated in RTCs, with suppression rates consistently exceeding 90% at 48–96 weeks (13, 14). In older PWH, the elimination of tenofovir and abacavir offers clinically meaningful renal and bone benefits, though proton pump inhibitor coprescription has emerged as the most common reason for ineligibility in real-world evaluations (15, 16). Predictors of failure include prior rilpivirine exposure, low CD4 nadir, and the food requirement for rilpivirine absorption, which may be challenging in patients with reduced appetite or dysphagia.

#### 3.2.3 PI-based dual regimens: real-world cohorts

Real-world data on DRV/r + 3TC and DRV/r + DTG generally support their virological efficacy as switch strategies in selected populations, with suppression rates comparable to those observed in randomized trials (17, 18). Observational cohorts have confirmed that these regimens are predominantly used in patients with prior INSTI exposure or intolerance, reflecting their guideline positioning as second-line dual options rather than preferred simplification strategies.

In older and comorbid PWH, real-world experience with PI-based dual regimens highlights significant practical limitations. The high drug–drug interaction potential of ritonavir and cobicistat boosting frequently necessitates dose adjustments or substitutions of concomitant medications, adding complexity to already challenging polypharmacy management. Gastrointestinal intolerance—including nausea, diarrhoea, and abdominal discomfort—represents a leading cause of discontinuation in older patients, in whom gastrointestinal comorbidities and nutritional vulnerability are more prevalent (19). Metabolic effects, including dyslipidaemia and insulin resistance, may further compound the cardiovascular risk burden in this population. Taken together, these real-world findings reinforce the limited role of PI-based dual regimens in older PWH and underscore the importance of careful patient selection and close monitoring when these regimens are used in the context of multimorbidity and polypharmacy.

## 4 Guideline-endorsed long-acting injectable dual therapy: evidence from RCTs and real-world studies

Long-acting injectable cabotegravir/rilpivirine (CAB/RPV) represents a paradigm shift in antiretroviral therapy, offering virologically suppressed adults a switch option that eliminates daily oral dosing entirely. Administered as two intramuscular injections—one of cabotegravir, an integrase strand transfer inhibitor, and one of rilpivirine, a non-nucleoside reverse transcriptase inhibitor—every four or eight weeks, CAB/RPV is the first long-acting injectable ART regimen endorsed by international guidelines as a switch strategy in eligible individuals. Beyond its virological rationale, CAB/RPV addresses dimensions of HIV care that extend beyond pharmacological efficacy, including treatment fatigue, stigma associated with daily pill-taking, and the preservation of treatment privacy. These features may carry particular relevance in older PWH, in whom adherence challenges, polypharmacy burden, and quality-of-life considerations increasingly shape therapeutic decisions.

### 4.1 Evidence from randomized controlled trials

The pivotal evidence for CAB/RPV was established by the ATLAS and FLAIR trials, two phase III open-label randomized studies that evaluated monthly (every four weeks, Q4W) CAB/RPV injections against continued oral ART in virologically suppressed adults. In ATLAS, participants were switched from any stable three-drug oral regimen to CAB/RPV Q4W or maintained their current therapy. At 48 weeks, CAB/RPV demonstrated non-inferiority to continued oral ART, with virological suppression rates of approximately 92% in both arms (20). FLAIR similarly randomized treatment-naive individuals who had achieved virological suppression on a dolutegravir-based induction regimen to either CAB/RPV Q4W or continued oral therapy, with comparable results at 48 and 96 weeks (21–23).

Both trials reported low rates of confirmed virological failure. However, a small but clinically significant subset of participants developed treatment-emergent resistance to rilpivirine and, less frequently, to cabotegravir. *post-hoc* analyses identified subtherapeutic drug exposure, baseline rilpivirine resistance-associated mutations, and HIV-1 subtype A6/A1 infection as key risk factors for virological failure, informing the eligibility criteria subsequently incorporated into clinical guidelines (24). With respect to older PWH, both trials included participants aged ≥50 years, and subgroup analyses generally demonstrated consistent virological outcomes across age strata, although the proportion of older individuals remained relatively small and those with significant comorbidities or complex drug interaction profiles were largely underrepresented (23).

The ATLAS-2M trial addressed the question of whether a less frequent dosing interval could maintain virological efficacy, randomizing virologically suppressed adults already receiving or initiating CAB/RPV to either every-eight-week (Q8W) or every-four-week (Q4W) injections. At 48 weeks, Q8W dosing demonstrated non-inferiority to Q4W dosing, with virological suppression rates exceeding 94% in both arms (22). These results were sustained at 96 weeks, with no significant difference in the rate of confirmed virological failure between dosing intervals (23).

The approval of Q8W dosing represents a clinically meaningful advance, reducing the number of clinic visits required for injection administration and potentially improving quality of life and treatment sustainability. In older PWH, the Q8W schedule may be particularly advantageous by decreasing the frequency of travel to healthcare facilities, a relevant consideration in individuals with mobility limitations, transportation barriers, or significant functional decline. At the same time, the Q8W interval requires careful patient selection, as delayed recognition of tolerability issues or early virological rebound may have greater consequences when monitoring intervals are extended. Subgroup analyses from ATLAS-2M did not reveal differential efficacy by age, though the same limitations regarding underrepresentation of older and comorbid individuals apply (22).

The CABINET trial extended the evidence base for CAB/RPV to a population not previously studied in pivotal trials: virologically suppressed individuals with a history of virological failure on prior ART regimens, provided they had no documented resistance to cabotegravir or rilpivirine. This population is clinically relevant, as many long-term PWH—particularly older individuals with extensive treatment histories predating the era of modern high-barrier regimens—have experienced at least one episode of virological failure.

At 48 weeks, CABINET demonstrated non-inferiority of CAB/RPV Q4W to continued oral ART in this historically more challenging population, with virological suppression rates comparable to those observed in ATLAS and FLAIR]. The rate of confirmed virological failure with emergent resistance remained low, supporting the conclusion that a history of prior virological failure does not *per se* preclude successful use of CAB/RPV, provided resistance testing confirms the absence of relevant mutations. These findings are particularly pertinent for older PWH, who disproportionately carry histories of prior treatment failure from the era of suboptimal regimens, and for whom CABINET data provide reassurance that long-acting injectable therapy remains a viable option following thorough resistance evaluation.

### 4.2 Real-world evidence

Since its regulatory approval and guideline endorsement, real-world experience with CAB/RPV has expanded across multiple healthcare settings, providing important complementary data on effectiveness, tolerability, and patient-reported outcomes in populations that more closely reflect routine clinical practice. Observational cohorts have generally confirmed the high rates of virological suppression observed in pivotal trials, with reported suppression rates at 48 weeks ranging from 90 to 96% across diverse patient populations (25–27).

Real-world studies have also shed light on the implementation challenges associated with the injectable route of administration. Injection site reactions—the most frequently reported adverse event in both trials and observational studies—have been identified as a leading cause of early discontinuation in a subset of patients, though severe reactions are uncommon, and most individuals tolerate the injections well over time (28, 29). Logistical barriers, including the requirement for clinic-based administration, cold-chain storage, and coordination of injection appointments, have emerged as real-world determinants of treatment continuity, particularly in settings with limited healthcare infrastructure or in patients with poor engagement in care.

In older and comorbid PWH, real-world data on CAB/RPV are still accumulating, but early findings suggest that this population may derive particular benefit from the elimination of daily oral dosing, especially in the context of high pill burden, treatment fatigue, or cognitive impairment affecting medication management (30). Several cohort analyses have reported that motivations for switching to CAB/RPV in older individuals frequently include simplification of the oral regimen, avoidance of drug–drug interactions with concomitant medications, and patient preference for injection-based therapy. Virological outcomes in older PWH switching to CAB/RPV have been broadly consistent with those in younger patients, though the number of individuals aged ≥65 years in published real-world series remains limited.

Comprehensive medication review prior to initiation is therefore essential, and real-world experience highlights the value of multidisciplinary input—including pharmacist involvement—when evaluating older candidates for long-acting injectable therapy.

Emerging real-world data have also begun to characterize predictors of virological failure with CAB/RPV outside controlled trial settings. Consistent with trial-based findings, subtherapeutic drug levels—potentially related to high body mass index affecting intramuscular drug absorption or to delayed or missed injection appointments—have been associated with increased risk of virological rebound in observational cohorts (31). In older PWH, additional considerations include the potential impact of sarcopenia and reduced muscle mass on intramuscular drug absorption, a pharmacokinetic variable that has received limited systematic attention in this population and warrants further investigation.

Taken together, evidence from the ATLAS, FLAIR, ATLAS-2M, and CABINET trials, supported by growing real-world experience, establishes CAB/RPV as an effective and well-tolerated switch strategy in appropriately selected virologically suppressed adults. In older and comorbid PWH, the potential benefits of eliminating daily oral therapy must be carefully weighed against eligibility constraints—particularly the rilpivirine interaction profile and the absence of hepatitis B activity—and the practical demands of clinic-based injectable administration. Patient preference, functional status, and the broader geriatric profile of the individual should be central to the decision to switch to long-acting injectable therapy in this population.

## 5 Real-world evidence and the impact of aging and comorbidities

Real-world evidence (RWE) plays a critical role in complementing data derived from RCTs by capturing the effectiveness and safety of ART in broader and more heterogeneous patient populations. In contrast to RCTs, observational studies routinely include individuals who are underrepresented or excluded from clinical trials, such as older people, those with long-standing infection, multiple comorbidities, prior treatment failures, or complex therapeutic histories.

Across multiple real-world cohorts, dual ART has demonstrated high rates of virological suppression, often exceeding 90%–95%, including in individuals with more complex clinical profiles. These findings support the external validity of RCT results and suggest that dual therapy can be effectively implemented in routine clinical practice. At the same time, real-world studies have identified predictors of virological rebound that may be less apparent in controlled trial settings. These include low nadir CD4 cell count, extensive prior antiretroviral exposure, archived resistance mutations, suboptimal adherence, and markers of frailty, underscoring the importance of careful patient selection and appropriate monitoring when treatment simplification is considered outside controlled environments (18).

The demographic shift toward an aging HIV population represents a major driver of ART complexity. As PWH age, they experience a higher prevalence of non-AIDS comorbidities, including cardiovascular disease, chronic kidney disease, diabetes mellitus, dyslipidaemia, osteoporosis, and neurocognitive disorders. Management of these conditions frequently requires multiple concomitant medications, resulting in polypharmacy and an increased risk of drug–drug interactions and cumulative toxicity (19). In this context, regimen simplification—including a reduction in the number of antiretroviral agents—has emerged as a potential strategy not only to maintain virological suppression but also to improve long-term tolerability, safety, and quality of life. Observational data from Italian cohorts provide a pragmatic illustration of these dynamics. In the GEPPO (Geriatric Patients Living with HIV Observational) cohort, which included PWH aged ≥65 years, approximately one quarter of patients were receiving simplified regimens, including dual therapy. In this setting, multimorbidity and polypharmacy emerged as key drivers of treatment choice, often outweighing purely virological or immunological considerations, while virological suppression was largely maintained (14).

Immunological outcomes in aging PWH may differ from those observed in younger populations. Several real-world studies have reported slower and less pronounced CD4 cell recovery in individuals with long-standing HIV infection and low nadir CD4 counts, even in the presence of sustained virological suppression. In addition, normalization of the CD4/CD8 ratio appears less frequent in older patients, reflecting persistent immune activation, immunosenescence, and chronic inflammation. These alterations have been associated with an increased risk of non-AIDS morbidity and mortality and may contribute to the residual clinical vulnerability observed in virologically suppressed older PWH (20).

Taken together, these findings suggest that, in aging populations, decisions regarding dual therapy should be based on an individualized risk–benefit assessment extending beyond virological efficacy alone. Immunological history, frailty, inflammatory burden, comorbidities, and concomitant medications should all be considered. In this setting, RWE provides important complementary insights to guide antiretroviral strategy selection in an increasingly older and clinically complex HIV population.

## 6 Bridging trials and real life: why outcomes differ

Discrepancies between outcomes observed in RCTs and those reported in real-world studies largely reflect differences in study design, patient selection, and healthcare delivery contexts. By design, RCTs prioritize internal validity and typically enroll selected individuals with stable virological suppression, limited comorbidity burden, preserved organ function, and a high likelihood of adherence. In addition, trial participants benefit from frequent follow-up visits, structured adherence support, protocol-driven monitoring, and timely management of adverse events. Collectively, these features optimize treatment outcomes under controlled conditions and may not fully reflect treatment effectiveness when compared with routine clinical practice (21).

In contrast, real-world observational cohorts capture the full heterogeneity of PWH including individuals with frailty, multimorbidity, psychiatric comorbidities, substance use disorders, socioeconomic vulnerability, and inconsistent engagement in care. In these settings, variability in adherence, delayed recognition of treatment-related toxicity, and less intensive monitoring are more common and may substantially influence both virological and clinical outcomes. Moreover, differences in healthcare system organization—such as access to specialized HIV care, availability of multidisciplinary teams, and continuity of follow-up—play a critical role in shaping real-life effectiveness and contribute to the gap between trial efficacy and observational effectiveness.

The key methodological and contextual differences between randomized controlled trials and real-world observational studies that contribute to these discrepancies are summarized in Table 1. These methodological and system-level differences are particularly relevant in aging populations. Older PWH are more likely to present with multiple chronic comorbidities and to be exposed to polypharmacy, increasing the risk of drug–drug interactions and cumulative toxicity. Age-related cognitive decline, functional limitations, and sensory impairment may further compromise adherence and self-management. Importantly, these patient-level vulnerabilities may interact with healthcare system constraints and trial design features, amplifying discrepancies between outcomes observed in controlled trials and those achieved in routine care (23).

**Table 1:** Eligibility criteria and contraindications for guideline-endorsed dual antiretroviral regimens.

Clinical parameter	DTG/3TC	DTG/RPV	CAB/RPV (injectable)	DRV/r or DRV/c + 3TC^†^	DRV/r + DTG^†^
Eligibility criteria					
Indication	Naive + switch	Switch only	Switch only	Switch only	Switch only
Virological suppression required	Yes (switch); No (naive)	Yes	Yes	Yes	Yes
Prior failure/resistance	No prior failure on 3TC/FTC; no DTG resistance	No prior failure on NNRTI or INSTI	No prior failure on NNRTI or INSTI	No prior failure on 3TC; no PI resistance	No INSTI or PI resistance
Hepatitis B coinfection	Contraindicated	Contraindicated	Contraindicated	Contraindicated	Contraindicated
Key contraindications and restrictions					
Proton pump inhibitors	No restriction	Contraindicated	Contraindicated	No restriction	No restriction
Anticonvulsants (enzyme-inducing)	Avoid	Contraindicated	Contraindicated	Contraindicated	Avoid
eGFR < 30 mL/min	Caution (3TC dose adjustment)	Caution	No renal adjustment required	Caution (3TC dose adjustment)	Caution
Food requirement	None	≥ 500 kcal required	None (injectable)	With food (PI absorption)	With food (PI absorption)
Drug–drug interaction potential	Low	Moderate	Moderate (RPV)	High (boosted PI)	High (boosted PI)

† PI-based dual regimens (DRV/r or DRV/c + 3TC; DRV/r + DTG) are highlighted in grey and are not guideline-endorsed as preferred simplification strategies; they are generally reserved for patients with intolerance or resistance to integrase strand transfer inhibitors. Eligibility and contraindication profiles based on current EACS and DHHS guidelines.

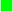
 Green: favourable/no restriction 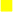 Amber: caution/use with monitoring 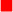 Red: contraindicated/avoid.

In this context, interpretation of RCT data—especially when informing treatment simplification strategies such as dual ART—requires careful consideration of real-world applicability. While RCTs establish efficacy under optimal conditions, real-world evidence provides important insight into how these strategies perform in everyday clinical practice, especially among older and more clinically vulnerable populations. By identifying patient- and system-level determinants of treatment success or failure, observational studies support individualized decision-making and risk stratification.

This gap between trial efficacy and real-world effectiveness fuels two complementary clinical perspectives. One emphasizes the potential benefits of reducing long-term drug exposure and interaction burden through simplification, particularly in aging patients with multimorbidity. The other prioritizes maximal virological robustness and “forgiveness,” suggesting that older and frail individuals may benefit from the higher resilience of three-drug regimens when adherence is imperfect or when archived resistance cannot be confidently excluded.

Ultimately, bridging the gap between trials and real life is essential for optimizing long-term HIV management. Integrating evidence from randomized trials with real-world data enables clinicians to contextualize efficacy results, anticipate challenges related to adherence and tolerability, and tailor antiretroviral strategies to the evolving needs of aging PWH. In this sense, real-world evidence is not merely complementary but indispensable for translating clinical trial findings into effective, patient-centred care. In aging populations, the clinical relevance of ART efficacy must therefore be interpreted not only in terms of virological outcomes, but also through the lens of feasibility, sustainability, and long-term clinical resilience.

## 7 Selecting the right dual regimen in older and comorbid PWH: from evidence to clinical practice

Integrated evidence from RCTs and real-world studies supports the use of dual antiretroviral therapy as a viable option in carefully selected patients. From a clinical perspective, however, dual therapy should not be viewed as a purely virologically driven simplification strategy. Rather, it represents an individualized treatment approach requiring a balance between efficacy, long-term safety, comorbidities, polypharmacy, and patient-centred outcomes.

The eligibility criteria and key contraindications for each guideline-endorsed dual regimen are summarized in Table 1. Overall, the most suitable candidates for dual therapy are individuals with sustained virological suppression, no documented history of virological failure involving agents included in the dual regimen, and no evidence of archived resistance mutations that could compromise treatment efficacy. The absence of chronic hepatitis B virus coinfection remains an important prerequisite for most dual regimens, given the lack of HBV activity and the associated risk of viral reactivation. Reliable and sustained adherence is also a key consideration, as dual therapy generally offers a lower pharmacological “forgiveness” margin compared with standard three-drug regimens (22).

Practical considerations relevant to the selection of dual therapy in older and comorbid PWH are summarized in Table 2. Patient selection in this population requires a broader and more nuanced clinical assessment. Renal function should be carefully evaluated, particularly in the context of age-related decline and concomitant nephrotoxic medications. Hepatic function assessment may also be relevant, especially in individuals with metabolic comorbidities or prior liver disease. Cardiovascular risk stratification is essential, as aging PWH frequently present with a high burden of both traditional and HIV-related cardiovascular risk factors (6, 19). The potential for clinically significant drug–drug interactions should be systematically reviewed, particularly in the setting of polypharmacy, where interactions involving statins, anticoagulants, antidiabetic agents, and psychotropic medications are common (14). Beyond organ-specific considerations, markers of frailty, functional capacity, and cognitive performance may substantially influence both tolerability and adherence and are therefore increasingly relevant to therapeutic decision-making³.

**Table 2:** Practical considerations for dual antiretroviral therapy selection in older and comorbid people with HIV.

**Clinical domain**	**DTG/3TC**	**DTG/RPV**	**CAB/RPV (injectable)**
Toxicity profile			
Renal safety	Favourable (no TDF/ABC)	Favourable (no TDF/ABC)	Favourable (no TDF/ABC)
Bone safety	Favourable (no TDF)	Favourable (no TDF)	Favourable (no TDF)
Cardiovascular safety	Favourable (no ABC)	Favourable (no ABC)	Favourable (no ABC)
Metabolic profile	Favourable vs boosted PI	Favourable vs boosted PI	Favourable vs boosted PI
Neuropsychiatric effects	Insomnia reported; monitor in older patients	Generally well tolerated	Generally well tolerated; injection site reactions
Drug–drug interactions in older PWH			
Cardiometabolic medications	Low interaction risk	Moderate risk (RPV interactions)	Moderate risk (RPV interactions)
Anticoagulants	Low interaction risk	Monitor (RPV interactions)	Monitor (RPV interactions)
Antacids/PPIs	No restriction	PPIs contraindicated	PPIs contraindicated
Adherence and practical considerations			
Pill burden	1 tablet once daily	1 tablet once daily	No daily pills required
Cognitive impairment/treatment fatigue	May be challenging with daily dosing	Food requirement adds complexity	Advantageous: removes daily self-management
Mobility/access to clinic	No clinic visits required for dosing	No clinic visits required for dosing	Regular clinic attendance required; barrier in frail patients
Sarcopenia/reduced muscle mass	Not applicable	Not applicable	May affect IM absorption; limited data
Overall profile in older/comorbid PWH			
Preferred when	High pill burden; renal/bone concerns; no HBV; no prior 3TC failure	No PPI use; adequate nutrition; no prior NNRTI failure	Treatment fatigue; cognitive impairment; high pill burden; good mobility
Caution or avoid when	HBV coinfection; prior 3TC/FTC failure; documented resistance	PPI use; malnutrition; prior NNRTI failure; enzyme-inducing drugs	Poor mobility; missed appointments; PPI use; sarcopenia; HBV

Table 2 refers to guideline-endorsed integrase-based dual regimens (DTG/3TC, DTG/RPV, CAB/RPV). PI-based dual regimens (DRV/r + 3TC, DRV/r + DTG) are not included given their more limited use in older PWH; their key practical limitations in this population—including high drug–drug interaction potential with boosted PI boosting, gastrointestinal intolerance, and unfavourable metabolic profile—are discussed in Section 2.2.3.

▪ Green: favourable/no restriction ▪ Amber: caution/use with monitoring ▪ Red: contraindicated/avoid.

DTG, dolutegravir; 3TC, lamivudine; RPV, rilpivirine; CAB, cabotegravir; DRV, darunavir; r, ritonavir; c, cobicistat; TDF, tenofovir disoproxil fumarate; ABC, abacavir; INSTI, integrase strand transfer inhibitor; NNRTI, non-nucleoside reverse transcriptase inhibitor; PI, protease inhibitor; HBV, hepatitis B virus; PPI, proton pump inhibitor; eGFR, estimated glomerular filtration rate; IM, intramuscular; Q4W, every four weeks; Q8W, every eight weeks. 2DR: Two Drug R*e*gimen; 3DR: Three Drug Regimen; RCT: Randomized Clinical Trials; RWE: Real world evidence; HBV: Hepatitis B Virus; TDF: Tenofovir Disoproxil Fumarate; DDI: Drug-Drug Interaction; PK/PD: Pharmacokinetic/Pharmacodynamic.

Patient preference and quality-of-life considerations should also play a central role in the selection process. Simplification to dual therapy may reduce pill burden, cumulative toxicity, and treatment fatigue, thereby improving treatment satisfaction and long-term adherence. Shared decision-making, supported by a transparent discussion of potential benefits and risks, is particularly important in older patients, for whom preserving functional independence and minimizing adverse events may be as clinically meaningful as achieving virological outcomes.

Conversely, certain clinical profiles warrant a more conservative approach. Individuals with a history of virological failure—particularly on regimens containing agents with a low genetic barrier to resistance—may be at increased risk of virological rebound following a switch to dual therapy (10). Patients with complex immunological profiles, such as very low nadir CD4 cell counts, suboptimal immune recovery despite prolonged viral suppression, or persistently inverted CD4/CD8 ratios, may derive greater benefit from the virological robustness of three-drug regimens (20). Similarly, individuals with advanced immunosuppression, ongoing immune activation, or unstable engagement in care may not represent optimal candidates for treatment simplification (10).

In these higher-risk scenarios, maintaining a standard three-drug regimen may provide greater virological resilience. If treatment simplification is nevertheless considered, it should be approached cautiously, with close monitoring of viral load, adherence, and tolerability, particularly during the early phases following the switch. Overall, individualized risk stratification—guided by the clinical framework outlined in Tables 1, 2—remains essential to maximize the potential benefits of dual therapy while minimizing the risk of virological failure or clinical deterioration.

## 4 Discussion

The choice between dual and three-drug antiretroviral therapy in aging and comorbid people with HIV should be interpreted within a broader framework that balances durable viral suppression with long-term safety, feasibility, and patient-centred outcomes. In older adults, regimen selection is rarely driven by virological endpoints alone; rather, it is shaped by multimorbidity, polypharmacy, organ-function, and the need to preserve functional status and quality of life over time.

A central consideration is the balance between treatment simplification and virological “forgiveness.” While randomized trials support the non-inferiority of selected two-drug regimens under controlled conditions, real-world practice includes patients with frailty, cognitive impairment, inconsistent engagement in care, and complex treatment histories. In these contexts, clinicians may reasonably adopt different strategies, weighing the potential benefits of simplification against the robustness of three-drug regimens, particularly when historical resistance data are incomplete or adherence may be variable.

A critical limitation of the current evidence base is the persistent underrepresentation of older and frail PWH in randomized controlled trials. Aging individuals—particularly those aged ≥65 years with significant multimorbidity, polypharmacy, or functional impairment—are frequently excluded from pivotal studies by design, limiting the generalizability of trial results to the very population most likely to benefit from reduced toxicity and interaction burden. Where older participants are included, they typically represent a minority of the enrolled population and are rarely analysed as a primary stratum. This structural gap between trial populations and real-world patients undermines the direct applicability of efficacy data and highlights the urgent need for randomized studies that enrol older and comorbid PWH as the primary target population, rather than as a subgroup of convenience.

Beyond virological endpoints, most available studies fail to capture outcomes that are particularly meaningful to older adults. In aging PWH, clinically relevant endpoints extend well beyond viral suppression to include functional status, risk of falls, cognitive trajectories, treatment burden, and health-related quality of life—dimensions that may significantly influence the net clinical benefit of different antiretroviral strategies. Treatment simplification may offer advantages not only by reducing drug exposure and drug–drug interactions, but also by improving tolerability, minimizing treatment burden, and supporting adherence in patients with frailty or cognitive impairment. However, robust data linking antiretroviral strategies to these geriatric-relevant outcomes remain scarce, and long-term follow-up data extending beyond 96–144 weeks are limited across all dual therapy trials. Future studies should prioritize the systematic integration of geriatric assessment tools and patient-reported outcome measures into both trial design and clinical practice, to better capture the full spectrum of treatment impact in this population.

Looking ahead, several developments hold promise for advancing the field. The expansion of long-acting injectable strategies may offer advantages in older PWH with high pill burden or treatment fatigue, provided that feasibility considerations—including mobility, clinic access, and intramuscular drug absorption in the context of sarcopenia—are carefully addressed. More broadly, the adoption of flexible, individualized treatment pathways that allow regimen complexity to evolve over time in response to aging, emerging comorbidities, and shifting patient preferences represents an important clinical and research priority. Ultimately, optimizing antiretroviral therapy in older PWH requires moving beyond a one-size-fits-all approach, toward a geriatric-informed model of HIV care in which treatment decisions are guided by the full clinical and functional profile of the individual.

## 5 Conclusions

Dual antiretroviral therapy has emerged as a validated option in selected clinical scenarios, with randomized controlled trials demonstrating non-inferior virological efficacy for guideline-endorsed regimens under controlled conditions (11–13, 17, 23). Real-world observational studies extend these findings to more heterogeneous populations, supporting the feasibility of dual therapy in routine clinical practice while identifying factors that may limit the success of simplification—including multimorbidity, polypharmacy, frailty, immunological vulnerability, and suboptimal adherence (14, 18, 24). The potential benefits of dual regimens extend beyond virological control to encompass improved tolerability, reduced drug–drug interaction burden, and patient-centred outcomes that are increasingly relevant in the context of aging HIV care.

However, the role of dual therapy in older and multimorbid PWH remains incompletely defined. These individuals are underrepresented in pivotal trials, and long-term data on geriatric-relevant outcomes—including functional status, cognitive trajectories, and health-related quality of life—are scarce. Dedicated studies enrolling older PWH as the primary target population, with integration of geriatric assessment and patient-reported outcome measures, are urgently needed to fill this evidence gap.

No single antiretroviral strategy can be considered optimal for all aging PWH. The choice between dual and three-drug regimens should be tailored within a flexible, longitudinal treatment framework that accounts for virological history, immunological status, organ function, comorbidities, functional capacity, and patient preferences—guided by the principles of geriatric medicine rather than virological endpoints alone.
